# Heat-Polymerized Resin Containing Dimethylaminododecyl Methacrylate Inhibits *Candida albicans* Biofilm

**DOI:** 10.3390/ma10040431

**Published:** 2017-04-20

**Authors:** Hui Chen, Qi Han, Xuedong Zhou, Keke Zhang, Suping Wang, Hockin H. K. Xu, Michael D. Weir, Mingye Feng, Mingyun Li, Xian Peng, Biao Ren, Lei Cheng

**Affiliations:** 1 State Key Laboratory of Oral Diseases, Sichuan University, Chengdu 610041, China; chenhui19910125@126.com (H.C.); hanqi992011@163.com (Q.H.); zhouxd@scu.edu.cn (X.Z.); zalmancoco@163.com (K.Z.); wangsupingdent@163.com (S.W.); fengmingye@gmail.com (M.F.); limingyun@scu.edu.cn (M.L.); pengx@scu.edu.cn (X.P.); 2 Department of Operative Dentistry and Endodontics, West China Hospital of Stomatology, Sichuan University, Chengdu 610041, China; 3 Department of Preventive Dentistry, Stomatological Hospital of Chongqing Medical University, Chongqing 401147, China; 4 Biomaterials & Tissue Engineering Division, Department of Endodontics, Prosthodontics and Operative Dentistry, University of Maryland Dental School, Baltimore, MD 21201, USA; hxu@umaryland.edu (H.H.K.X.); MWeir@umaryland.edu (M.D.W.)

**Keywords:** heat-polymerized resin, dimethylaminododecyl methacrylate, *Candida albicans* biofilms, denture stomatitis, antifungal properties.

## Abstract

The prevalence of stomatitis, especially caused by *Candida albicans*, has highlighted the need of new antifungal denture materials. This study aimed to develop an antifungal heat-curing resin containing quaternary ammonium monomer (dimethylaminododecyl methacrylate, DMADDM), and evaluate its physical performance and antifungal properties. The discs were prepared by incorporating DMADDM into the polymer liquid of a methyl methacrylate-based, heat-polymerizing resin at 0% (control), 5%, 10%, and 20% (*w*/*w*). Flexure strength, bond quality, surface charge density, and surface roughness were measured to evaluate the physical properties of resin. The specimens were incubated with *C. albicans* solution in medium to form biofilms. Then Colony-Forming Units, XTT assay, and scanning electron microscope were used to evaluate antifungal effect of DMADDM-modified resin. DMADDM modified acrylic resin had no effect on the flexural strength, bond quality, and surface roughness, but it increased the surface charge density significantly. Meanwhile, this new resin inhibited the *C. albicans* biofilm significantly according to the XTT assay and CFU counting. The hyphae in *C. albicans* biofilm also reduced in DMADDM-containing groups observed by SEM. DMADDM modified acrylic resin was effective in the inhibition of *C. albicans* biofilm with good physical properties.

## 1. Introduction

Heat-curing resins are frequently used in prosthodontics, particularly in complete denture bases and partial denture bases due to their esthetically acceptable color and availability at low cost. However, there is a high incidence of denture-induced stomatitis in denture wearers [1,2,3].

*Candida* species, especially *Candida albicans*, have a high detection rates in the denture stomatitis [4]. The association between *C. albicans* and denture stomatitis has been well documented [2,5,6,7]. *Candida*-associated denture stomatitis was observed in approximately 11% to 67% of otherwise healthy denture wearers [1]. *C. albicans* can exist in two basic forms, yeast phase and mycelial phase [8]. The switch between different forms of growth is one of the virulence factors, which can also lead to *Candida* biofilm formation [9,10]. Previous study indicated that the formation of *C. albicans* biofilms on dentures can not only assist the survival of fungal cells [5], but also increase the inflammation by secreting aspartic protease Sap4/Sap6, mycelium protein Hwp1, and adhesion gene *ALS3/EPA1* [11]. The biofilms of *C. albicans* are usually difficult to remove by mechanical or chemical cleaning compared to the planktonic cells [12,13]. Reducing *C. albicans* biofilm on the surface of the prosthesis is a pragmatic strategy to control denture stomatitis [3,14,15]. Although adequate denture cleaning is imperative for the prevention of denture stomatitis, it is more beneficial and necessary to develop an antifungal denture surface [3,14,15].

To grant the acrylic resin antifungal ability, a large number of antimicrobial agents were added into dental materials. All of them can be divided into two classes: released and non-released materials. For the released materials, early study indicated that tea tree oil and chlorhexidine gluconate were effective in inhibiting *C. albicans* growth on heat-polymerized acrylic resin and denture soft liner [13,16]. The denture base resin containing nano-silver showed antifungal activity and an inhibitory effect on adhesion and biofilm formation of *C. albicans*, especially at a higher concentration [17]. However, with the antimicrobial ingredients releasing, the mechanical properties will decrease and the antimicrobial property is unsustainable [12,14]. The biosecurity of released ingredients is also unclear [14,17,18,19,20].

Non-released antibacterial materials have been synthesized in many dental materials [21,22,23,24,25] and demonstrated good antibacterial effect. Quaternary ammonium methacrylates (QAMs), such as 12-Methacryloyloxydodecyl-pyridinium bromide (MDPB), can be copolymerized and covalently bonded in resins, immobilizing and exerting a contact-killing capability against oral bacteria and biofilms. Several other non-released antibacterial materials were recently reported, such as methacryloxylethylcetyl dimethylammoniumchloride (DMAE-CB) containing adhesive, antibacterial glass ionomer cements, antibacterial nanocomposites, and bonding agents using a quaternary ammoniumdimethacrylate (QADM). Dimethylaminododecyl methacrylate (DMADDM), a new kind of QAMs, was also added to composite resin, bonding agent, and glass-ionomer cement as a non-releasing agent, which has witnessed an antibacterial effect [25,26,27,28]. However, only a few articles described the QAMs (MDPB) as additives in acrylic resin to study the antibacterial activity of the new materials [29,30] and no antifungal investigation of the QAM-modified acrylic resin has been reported. Especially, the DMADDM has not been added in the acrylic resin to explore its antifungal effect [31,32,33].

The aim of this paper is to incorporate antibacterial DMADDM into heat-polymerized resin with a new process and further investigate the effects on both physical performance and the formation of *C. albicans* biofilms.

## 2. Materials and Methods

### 2.1. Synthesis of Antibacterial Monomer

Dimethylaminododecyl methacrylate (DMADDM) was synthesized according to a previously described process [27,34]. Briefly, 10 mmol of 2-(dimethylamino) ethyl methacrylate (DMAEMA), 10 mmol of 1-bromododecane (BDD), and 3 g of ethanol were mixed in a vial by capping and stirring at 70 °C for 24 h. Ethanol was evaporated after the reaction was completed. The clear liquid remaining in the vial was DMADDM, which can be verified via Fourier transform infrared spectroscopy.

### 2.2. Specimen Fabrication

**Figure 1:**
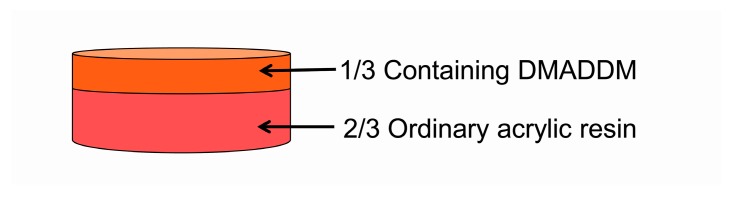
Double-decked acrylic resin containing DMADDM.

The commercial acrylic resin, Nature Cryl™ MC (GC America Inc., Alsip, IL, USA), was used for making samples. Acrylic resin was prepared via polymerizing heat-polymerizable powder and liquid following the manufacture instructions in a cavity die metal box (10 × 10 × 5 cm^3^). The acrylic resin was cut into small samples (11 × 11 × 3 mm^3^) by a diamond-coated band saw (Struers Minitom, Holstebro Kommune, Denmark). The control group was heat-polymerized with powder and liquid without DMADDM. We developed a new approach to making the double-decked acrylic resin (Figure 1). The double-decked acrylic resin can be manufactured as follows:

- DMADDM was added to heat-polymerizable liquid blending to a certain mass fraction (5%, 10%, and 20%).
- Untreated heat-polymerizable liquid was mixed with untreated powder, reacting until the paste stage.
- Treated heat-polymerizable liquid was mixed with untreated powder, reacting until the paste stage.
- One-third treated acrylic resin and two-thirds untreated acrylic resin in the lower, were placed into the upper and lower portions of cavity die box, respectively, and filled with gypsum at the same time (Figure 1). Pressure was used for polymerization by tightening bolts on the cavity die box and excess material was removed.
- The box was put into an incubator, reacting at 72 °C for 90 min and then at 100 °C for 60 min.
- Once the die box cooled down, acrylic resin was taken out and cut into certain size specimens (11 × 11 × 3 mm^3^) by a diamond-coated band saw (Struers Minitom).
- In turn, treated acrylic resin surface was polished with different particle of standard metallographic sandpaper (P400, P800, P1000, P1500, P2000, P2400, and P4000) (Struers Minitom).

After being polymerized, the upper third of samples showed slight loss of color. There was a natural color transition and no obvious dividing line from lower two-thirds to the upper third in the successful double-decked sample.

After immersion in distilled water at 37 °C for 24 h, the specimens were sterilized in an ethylene oxide sterilizer (Anprolene AN74i, Andersen, Haw River, NC, Germany). Specimens were separated into four groups: acrylic resin with 0% DMADDM; acrylic resin with 5% DMADDM; acrylic resin with 10% DMADDM; acrylic resin with 20% DMADDM. The first group was the control group while the others were the experimental groups.

### 2.3. Mechanical Testing

**Figure 2:**
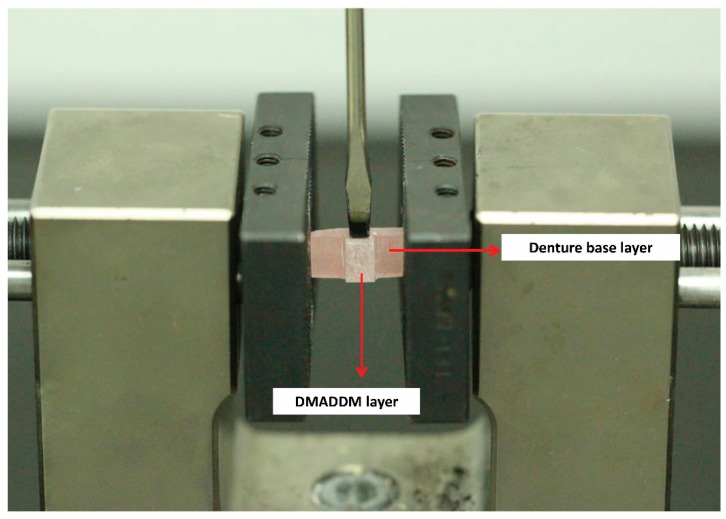
Sample clamped in Universal Testing Machine.

Bond quality test of the interface between two-thirds denture base layer and one-third DMADDM layer was tested with the help of Universal testing machine (5500R, MTS, Cary, NC, USA) [35,36]. The denture base layer was rigidly fixed to the holding arm of the machine. The DMADDM layer was on the middle and above the surface of the base layer. Shear force was applied with the help of a screwdriver perpendicular to the vertical axis of the DMADDM layer at a distance of 0.2 mm from the bond interface (Figure 2). The crosshead speed was 1 mm/min. The fracture faces were recorded and fracture strength was calculated.

Flexural strength of each acrylic resin specimen was measured via a three-point flexural test with a 15-mm span at a cross head-speed of 1 mm/min on a computer-controlled Universal Testing Machine (5500R, MTS, Cary, NC, USA). The flexural strength of the material was calculated by *S* = 3*P*_max_*L*/(2*bh*^2^), where *P*_max_ is the maximum load on the load-displacement curve, *L* is flexure span, *b* is specimen’s width, and *h* is specimen’s thickness [37,38].

### 2.4. Surface Roughness Observation

An AFM (Atomic Force Microscopy, 5500SPM, Agilent, Palo Alto, CA, USA) was used at high resolution with a sharp silicon tip (0.5 N/m) in tapping mode. The surface topography of the treated acrylic resin disk was obtained over an area 10 × 10 μm^2^. The surface roughness of the samples was provided with systemic software (SPIWIN 2.0, NSK, Tokyo, Japan) and data of Ra in different groups were compared [37,38].

### 2.5. Charge Density Testing

The charge density present on the polymer disk surfaces was quantified using a fluorescein dye method as previous study [39]. Acrylic resin disks were put in a 24-well plate. Fluorescein sodium salt (200 μL of 10 mg/mL) in deionized (DI) water was added into each well. Specimens were left in the dark at room temperature for 10 min. After removing the fluorescein solution and rinsing with DI water, each disk was placed in a new 24-well plate, and 200 μL of 0.1% (by mass) of cetyltrimethylammonium chloride (CTMAC) in DI water was added. Samples were shaken in the dark at room temperature for 20 min to absorb the bound dye. The CTMAC solution was supplemented with 10% (by volume) of 100 mM phosphate buffer at pH 8. Each sample’s absorbance was read at 501 nm via a plate reader (SpectraMax M5, Molecular Devices, Sunnyvale, CA, USA). The fluorescein concentration was calculated by Beers Law and an extinction coefficient of 77 mM^−1^·cm^−1^. Using a ratio of 1:1 for fluorescein molecules to the accessible quaternary ammonium groups, the surface charge density was calculated as the total molecules of charge per unit of exposed surface area. Six replicates were tested for each group.

### 2.6. Biofilm Formation Assay

*C. albicans* SC5314 (ATCC MYA-2876) were recovered on YPD plate (1% yeast extract, 2% peptone, 2% glucose, 1.5% agar) at 35 °C overnight. For the biofilm formation inhibition assay, the specimens were incubated with 2 mL of prepared *C. albicans* solution (final concentration: 1 × 10^5^ cells/mL) at 37 °C in spider medium (10 g nutrient broth, 10 g mannitol, and 2 g K_2_HPO_4_ dissolved in 1 L distilled water) for 120 h. After biofilm formation, non-adhering cells were removed by washing three times with autoclaved phosphate-buffered saline (PBS) [40]. All the experiments were repeated three times. The morphological structure and the biomass of biofilm will be tested in the following experiments.

### 2.7. C. albicans Biofilm Metabolic Activity and Biomass Assay

An XTT (2, 3-bis (2-methoxy-4-nitro-5-sulfo-phenyl)-2H-tetrazolium-5-caboxanilide) assay was used to determine the metabolic activity of the biofilm as described previously [41]. XTT/menadione assay mix was made from 12.5 XTT/menadione (*v*/*v*) using stock solutions of 1 mg/mL XTT (Invitrogen X6493, Carlsbad, CA, USA) dissolved in PBS and menadione (reagent grade; Nutritional Biochemicals Corp., Cleveland, OH, USA) dissolved in acetone (reagent grade). After biofilm formed on the disks, the discs were put in a 24-well plate (with PBS) to wash biofilms three times, removing non-adherent cells. The washed discs were placed in a new 24-well plate with 100 μL PBS containing 50 μL XTT/menadione solutions and incubated at 37 °C for 2 h in the dark. After incubation, 200 μL of the solution was transferred to a 96-well plate, and colorimetric changes in the solution were measured using a microplate reader (Chro Mate1, Awareness technology, Palm City, FL, USA) at 490 nm.

### 2.8. Biomass Calculation

Specimens with 120 h biofilms were transferred into tubes with 2 mL saline and the biofilm on each disk was harvested by sonication and vortexing (Fisher, Pittsburgh, PA, USA), and then serially diluted in saline. 100 μL final diluted cell suspension was spread on YPD agar plates and incubated at 37 °C for 24 h to recover the viable cells in the biofilms [40]. The colony forming units (CFU) were counted.

### 2.9. Observation of Biofilm Structure

Disk specimens with *C. albicans* incubated for 120 h were prepared for examination with scanning electron microscope (SEM) (Quanta 200, FEI Company, Hillsboro, OR, USA). Each specimen with adherent biofilm was rinsed with PBS, and then immersed in 1% glutaraldehyde in PBS at 4 °C for 4 h. The specimens were rinsed with PBS, subjected to graded ethanol dehydrations, and rinsed twice with 100% hexamethyldisilazane. The specimens were then sputter-coated with gold and examined via SEM [42].

### 2.10. Statistical Analysis

One-way analysis of variance (ANOVA) was performed to detect the significant effects of the variables; however, when the data were different ariances, Kruskal-Wallis test was used. A *p*-value *<* 0.05 was considered statistically significant.

## 3. Results

### 3.1. Physical Performance of Double-Decked Acrylic Resin

**Figure 3:**
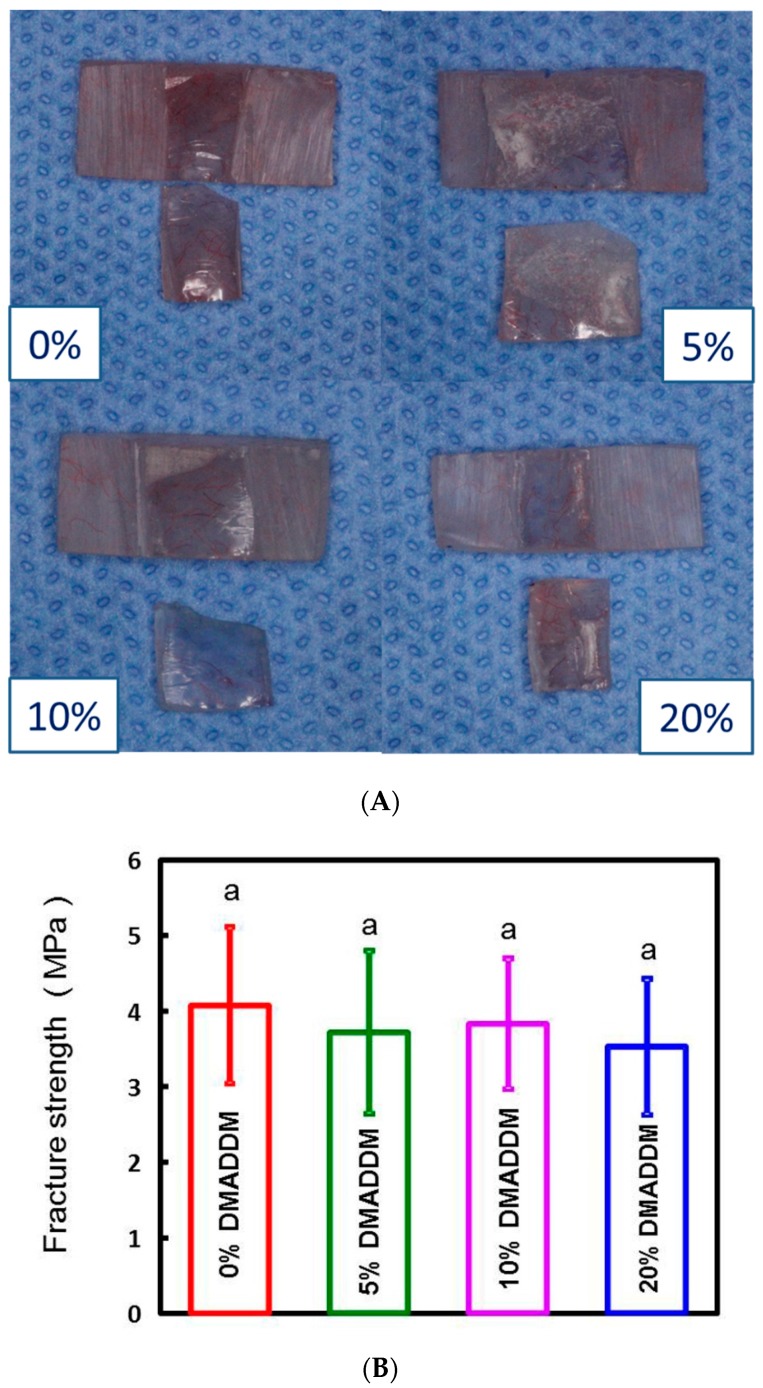
(**A**) The fractured face of different concentration of DMADDM samples in bond quality test; (**B**) The fracture strength of different DMADDM concentration samples. In each plot, the same letter indicates that there was no significant difference between the groups (*p* > 0.1).

**Figure 4:**
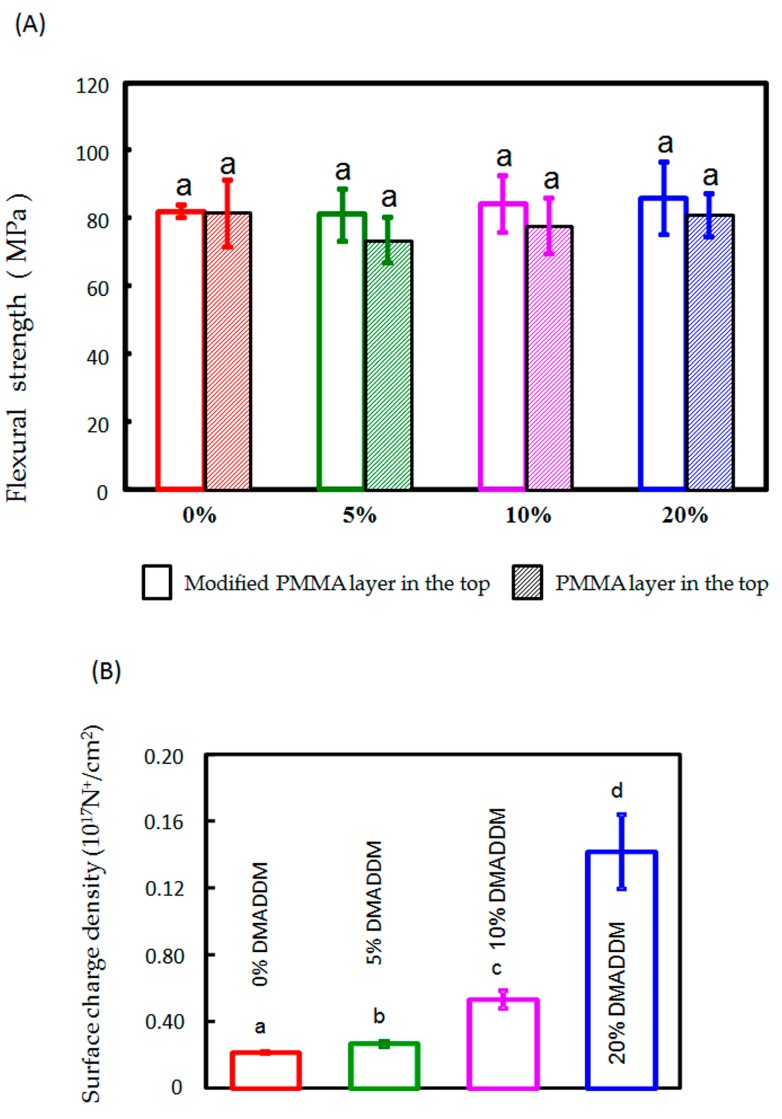
Flexural strength and surface charge density of double-decked acrylic resin. (**A**) Flexural strength. In each plot, the same letter indicates that there was no significant difference between the groups (*p* > 0.05); (**B**) Surface charge density. In each plot, dissimilar letters indicate values that are significantly different from each other (*p* < 0.05).

**Figure 5:**
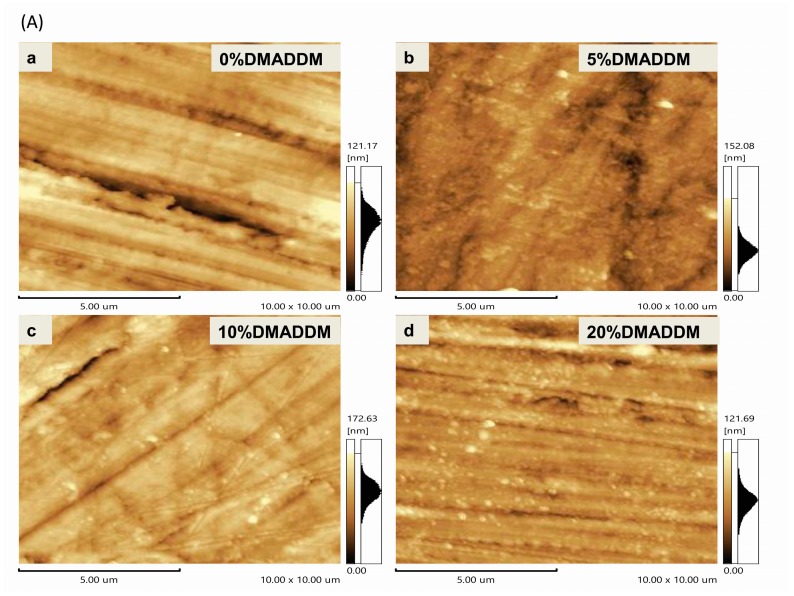

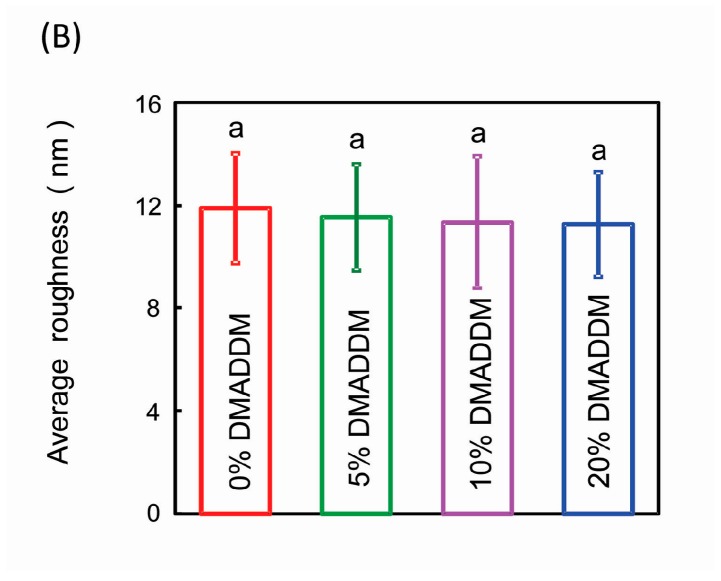
Surface roughness of different mass fraction of DMADDM resins. (**A**) **a**: The typical AFM image of a surface in the control group. **b**: The typical AFM image of a surface in the 5% DMADDM group. **c**: The typical AFM image of a surface in the 10% DMADDM group. **d**: The typical AFM image of a surface in the 20% DMADDM group; (**B**) The average roughness of acrylic resin. In each plot, similar letter indicates that there was no significant difference between the groups (*p* > 0.1).

After bond quality testing, the fractured faces of different DMADDM concentration samples were presented (Figure 3A). The fracture face of 0% DMADDM sample occurred in the mixed, the 5% DMADDM sample in the mixed, the 10% DMADDM sample in the mixed, the 20% DMADDM sample in the base resin layer respectively. None of the fracture faces merely occurred in the adhesive interface between the two-thirds base layer and one-third DMADDM layer (Figure 3A). The acrylic resin with various DMADDM mass fractions (5%, 10%, and 20%) had fracture strength (Figure 3B) and flexural strength (Figure 4A) similar to that of the control group. Adding DMADDM into acrylic resin increased the surface charge density (Figure 4B) significantly. The charge density value of acrylic resin containing 20% DMADDM was about seven-times that of the control group. Meanwhile, the acrylic resin with various DMADDM mass fractions (5%, 10%, and 20%) had a similar surface roughness to that of the control group (Figure 5).

### 3.2. The Antifungal Properties of Double-Decked Acrylic Resin

**Figure 6:**
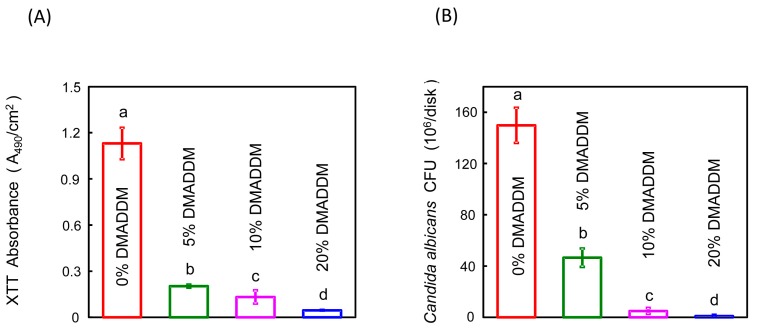
Biofilm metabolic and biomass analysis. (**A**) The XTT results of *C. albicans* biofilms formed on different DMADDM-containing denture bases. In each plot, dissimilar letters indicate values that are significantly different from each other (*p* < 0.05); (**B**) Colony-forming unit counts (CFU) of biofilms. The total CFU counts of 120 h of *C. albicans* in different dimethylaminododecyl methacrylate (DMADDM)-containing groups. In each plot, dissimilar letters indicate values that are significantly different from each other (*p* < 0.05).

**Figure 7:**
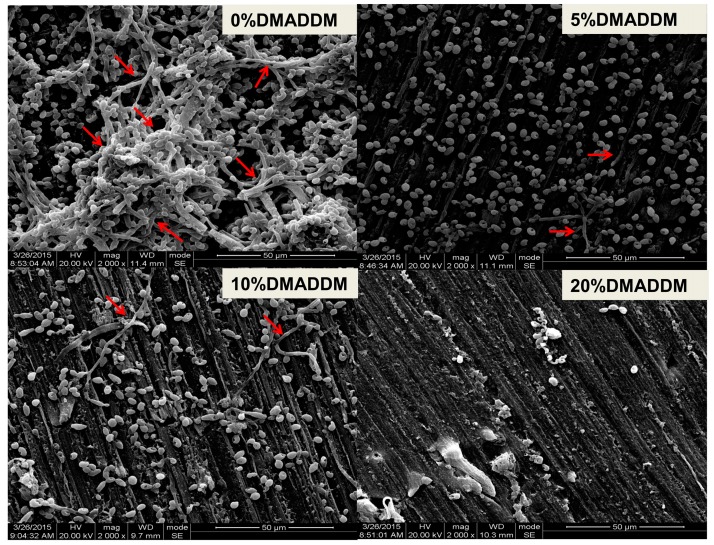
Scanning electron microscope (SEM) images of *C. albicans* biofilms. The red arrows indicate the hyphal form of *C. albicans*.

The XTT assay results showed that the DMADDM-modified samples had increased antimicrobial properties more significantly than the control group (Figure 6A). The results of CFU counting of different groups showed that the DMADDM containing group significantly inhibited the growth of *C. albicans* in biofilms in a dose-dependent manner compared to the control group (Figure 6B). The acrylic resin disks containing DMADDM had also reduced the biofilm on the surface at different concentrations of DMADDM compared with the control group according to the SEM observation (Figure 7). Importantly, the mycelium of *C. albicans* had decreased significantly in DMADDM-containing groups (Figure 7).

## 4. Discussion

There is a strong need of new dental materials that can inhibit *C. albicans* growth and reduce virulence due to the increase of denture stomatitis. In this study, we have developed a new method to synthesize the heat-polymerized acrylic resin containing DMADDM to grant the resin with antifungal ability. Recently, various coating approaches were applied to denture base materials with increased surface hydrophilicity to reduce *C. albicans* adherence [43,44,45]. Nevertheless, the coating layers were not stable [43,44,45] and were able to easily form rough surfaces to increase fungal adhesion [46]. Therefore, we developed a new double-decked resin as this article mentioned (Figure 1). This is an innovative approach to synthesize an antifungal resin. The upper one-third of the resin with DMADDM can maintain perfect antimicrobial effect and the two-thirds of substrate with ordinary resin can ensure decent mechanical properties. Bond quality test was used to measure the bond strength in the interface between the two-thirds denture base resin and the one-third DMADDM resin. None of the fracture faces occurred in the adhesive interface (Figure 3A). The result showed the fracture strength was similar to the control group (Figure 3B). Both methyl methacrylate composing the acrylic resin and DMADDM have double bonds in their molecular formula. Once the upper resin layer contacts the ordinary resin layer from paste stage, their double bonds will be opened and combined with each other to form an inseparable interface. After polymerization, there was a natural color transition and no obvious dividing line from the lower two-thirds to the upper third in the successful double-decked sample. The bond quality was allowed to utilize the strength of the materials. The double-decked acrylic resin was effective in the inhibition of *C. albicans* biofilm with good mechanical properties. Compared with the other methods, this way of manufacturing antifungal acrylic resin can be convenient and more effective. 

Physical properties are an important aspect of acrylic resin, the resins containing DMADDM (5%, 10%, and 20%) had no adverse effect on flexural strength compared to the control group in this double-decked model. The surface roughness of resin could influence fungal adhesion [47]. We polished double-decked samples to ensure that the roughness of resin would remain consistent before biofilm formation in the following tests. Further investigations are needed to confirm whether physical properties of acrylic resin containing DMADDM are affected by oral microenvironment and aging since this in vitro study was only executed in a short time.

*C. albicans* may grow in different types: yeast, pseudohyphal, and hyphal. The hyphal formation significantly decreased on the DMADDM-containing resin. The reason that DMADDM inhibited *C. albicans* filamentous growth may be that: (i) DMADDM eliminated the *C. albicans* cells directly; (ii) DMADDM took part in the inhibition of *C. albicans* hyphal development. We will identify the mechanism during the following investigation.

There were few reports on the antifungal mechanisms of QAM compared to the antibacterial mechanisms. Beyth N et al. indicated that the positively charged quaternary amine N^+^ of a QAM could attract the negatively-charged cell membrane of bacteria, disrupting the cell membrane and causing cytoplasmic leakage [48,49]. Similar to the bacteria, QAM can affect the fungal plasma membrane, causing mono- and divalent cation, as well as ATP, leakage, strongly disrupting plasma membrane structure and decreasing the survival of fungal cells [10,50,51]. In this study, the surface charge density of double-decked resin was increased (Figure 4). Therefore, the heat-polymerized resin with a higher concentration of DMADDM, which increased the positive charge density significantly, had stronger antifungal potency.

The hydrophobic surface could promote the adherence of *C. albicans* [52,53] and selectively increased the propensity of hyphal forms of *C. albicans* to colonize denture surfaces [54]. DMADDM has a hydrophilic group which modifies the surface of acrylic resin after polymerization, to reduce biofilm formation (adhesion) and hyphal development.

DMADDM is a kind of quaternary ammonium methacrylate (QAMs). It is indispensable for clinical application of materials with good biosafety. Previous studies showed human gingival fibroblasts and odontoblasts have good biocompatibility with DMADDM [39,46,55]. In an in vivo histological evaluation, Keke Zhang et al. proved that less than 20% DMADDM in denture material did not increase the inflammatory response, suggesting good biocompatibility and biosafety of the newly synthesized material containing DMADDM as an antimicrobial additive [56]. Moreover, Nurit Beytha et al. showed that the antimicrobial-compound QAMs were stable and did not leach out from material into the saliva. QAMs can cause stress not only to the cells with which they come into contact but also to the other outer cells in the surrounding environment. It was shown that bacterial lysis by QAM on the resin surface may function as a stressful condition, triggering programmed cell death (PCD) in the surrounding bacteria [57]. Han Zhou also found that resin containing DMADDM can kill the entire biofilm, not just the bacteria in contact. This outcome was consistent with Nurit Beytha [58]. Jin feng et al. added DMADDM to glass ionomer cement (GIC) and measured the release of DMADDM [28]. The result showed that the release of DMADDM could not be found in saliva [28]. Therefore, the double-decked acrylic resin is an antifungal material with good biosafety.

## 5. Conclusions

The current study developed a new double-decked acrylic resin containing DMADDM and investigated the material physical properties for the first time. Double-decked acrylic resin containing DMADDM was effective in the inhibition of *C. albicans* biofilm with good physical properties compared to the control group.
